# Temporal changes in mosquito abundance (*Culex pipiens*), avian malaria prevalence and lineage composition

**DOI:** 10.1186/1756-3305-6-307

**Published:** 2013-10-25

**Authors:** Fabrice Lalubin, Aline Delédevant, Olivier Glaizot, Philippe Christe

**Affiliations:** 1 Department of Ecology and Evolution, University of Lausanne, Lausanne CH-1015, Switzerland; 2 Museum of Zoology of Lausanne, Lausanne CH-1014, Switzerland

**Keywords:** *Culex pipiens*, *Plasmodium relictum*, *Plasmodium vaughani*, Temporal parasite community, Seasonality, Vector-borne disease

## Abstract

**Background:**

Knowledge on the temporal dynamics of host/vector/parasite interactions is a pre-requisite to further address relevant questions in the fields of epidemiology and evolutionary ecology of infectious diseases. In studies of avian malaria, the natural history of *Plasmodium* parasites with their natural mosquito vectors, however, is mostly unknown.

**Methods:**

Using artificial water containers placed in the field, we monitored the relative abundance of parous females of *Culex pipiens* mosquitoes during two years (2010–2011), in a population in western Switzerland. Additionally, we used molecular tools to examine changes in avian malaria prevalence and *Plasmodium* lineage composition in female *C*. *pipiens* caught throughout one field season (April-August) in 2011.

**Results:**

*C*. *pipiens* relative abundance varied both between years and months, and was associated with temperature fluctuations. Total *Plasmodium* prevalence was high and increased from spring to summer months (13.1-20.3%). The *Plasmodium* community was composed of seven different lineages including *P*. *relictum* (SGS1, GRW11 and PADOM02 lineages), *P*. *vaughani* (lineage SYAT05) and other *Plasmodium* spp. (AFTRU5, PADOM1, COLL1). The most prevalent lineages, *P*. *vaughani* (lineage SYAT05) and *P*. *relictum* (lineage SGS1), were consistently found between years, although they had antagonistic dominance patterns during the season survey.

**Conclusions:**

Our results suggest that the time window of analysis is critical in evaluating changes in the community of avian malaria lineages infecting mosquitoes. The potential determinants of the observed changes as well as their implications for future prospects on avian malaria are discussed.

## Background

Seasonal variations in ecological and climatic parameters such as day length, rainfall, temperature or available resources are particularly marked at mid-latitudes with temperate climates. Seasonality is highly important for the population dynamics of infectious diseases and often results in cyclic prevalence patterns of the parasites within susceptible host populations (reviewed in [1]). Cyclic dynamics may arise from seasonal modifications in the biology and the behaviour of animal hosts and their parasites favoring contact rates between them [2]. For instance, seasonal migration of animals may offer different hitchhiking trajectories for parasites and may shape the parasite community structure at a local scale [3].

Malaria parasites (*Plasmodium* spp., Haemosporidae: Apicomplexa) are extremely diversified protozoan blood parasites [4,5] that are transmitted to vertebrate hosts by blood-sucking dipteran insect vectors [6]. The general life cycle of *Plasmodium* parasites seems to be well conserved across vertebrate hosts [6,7], although their dynamics of infection within the vertebrate hosts can substantially vary depending on the combinations between host and parasite lineages e.g. [8-10]. Malaria-infected hosts classically suffer a first peak of parasitaemia (acute infection phase), which occurs about 15 days after the parasite inoculation. The parasite then gradually retreats from the blood to the host’s internal organs where it is no longer transmissible to the vectors (latent infection phase). The infection may remain latent for several months until a secondary blood relapse of the parasite arises. Cycles of latent infection and relapse can then reoccur at fixed time intervals.

Many studies have investigated the seasonal incidence of malaria parasites in susceptible host populations to further predict the risk of becoming infected [11]. Most of these longitudinal studies agree that malaria outbreaks generally arise synchronously in late spring or, in tropical zones, near the monsoon season [12-14]. This “spring relapse” has been particularly emphasized in avian malaria studies [15-22] and although it is believed to coincide with the seasonal peak abundance of the blood-sucking vectors [23], thus facilitating parasite transmission [24], the seasonal dynamics of major disease vectors remains understudied in temperate Europe [25].

The development of new PCR-based methods [26,27] has allowed the documentation of dynamic changes in the communities of avian *Plasmodium* lineages within wild bird species populations [28-32] or individual hosts [33-36]. Whilst seasonal changes in host immunocompetence could explain the observed patterns of abundance and persistence of avian *Plasmodium* lineages in these studies, we do not know much about the role of natural vectors in the epidemiology of avian malaria [37-39]. Recent epidemiological models have however demonstrated that they play a central role in *Plasmodium* temporal dynamics [40].

There is growing evidence that the northern house mosquito, *Culex pipiens* (Diptera: Culicidae), is a major vector of avian malaria in the northern hemisphere [41-47]. This mosquito, which can act as a vector of several other infectious diseases such as arboviruses [48], is sensitive to seasonal changes [49]. For instance, autumnal decreases in day length and temperature have been shown to trigger a genetic cascade [50] that inhibits host-seeking and blood-feeding behaviour in overwintering *C*. *pipiens* populations [51]. To get a better understanding of the complex malarial interactions, it is thus of crucial interest to account for the infection dynamics of the vectors, as well as their seasonal patterns of abundance.

Here, we monitored the relative abundance of one population of *C*. *pipiens* mosquitoes during two years (2010–2011) in western Switzerland. In 2011, we also surveyed this mosquito population for avian malaria infection from April to September. Our aims were (i) to investigate the relationship between climatic variables (rainfall and temperature) and mosquito population densities, (ii) to determine the *Plasmodium* infection dynamics of the vectors through the season and (iii) to document changes in the parasite community structure on a larger temporal scale, through data comparison with a previous long-term survey conducted at our study site on both mosquitoes and bird hosts. The present study is therefore part of a continuous effort to provide a better understanding of avian malaria interactions in a natural model system.

## Methods

### Study site and mosquito survey

Mosquito surveys were conducted from April to September 2010 and 2011, at the edge of the urban forest of Dorigny (46°31′N; 6°34′E; alt. 400 m), on the campus of the University of Lausanne (Switzerland). Temperature and precipitation data were obtained from the closest meteorological station (Swiss Federal Office of Meteorology and Climatology) located in Pully, about 7 km southeast of our study site. Rainfall collecting containers (50×30×25 cm) intended to provide gravid female mosquitoes with oviposition sites were set up at our spot survey in the early spring and removed in autumn. 160 to 179 containers were initially filled up with water from Lake Léman, located at the South of the study site, and baited with baker’s yeast so as to favour container visitation by gravid *Culex pipiens* females [52]. The containers were positioned one next to another, at a density of about 4 containers/m^2^. All containers were inspected twice a week for egg rafts. Because the number of collected egg rafts was strongly heterogeneous between the different containers, we measured *n*, the density of egg rafts, as the mean number of egg rafts collected per container per inspection date. Egg raft densities provided us with reliable estimates of the *C*. *pipiens* relative abundance throughout the year [53,54] and the measurements were congruent with the data gained from the survey of gravid *C*. *pipiens* with mosquito traps (see Additional file 1: Figure S1).

### Field-collection of adult gravid female mosquitoes

Collection of adult female *C*. *pipiens* was carried out two to three times per week from April to September 2011 (26 weeks), by using gravid mosquito traps (Bioquip, California). Each trapping day, gravid traps were set up at sunset on the containers that totaled the highest number of egg rafts during the preceding week. The traps were removed the next morning, after sunrise. Collected mosquitoes were transferred to individual plastic vials (SARTSDET, 30 ml) and were maintained unfed for 23 days on average, until they died. Freshly dead mosquitoes were transferred within the day to -80°C to further determine their malaria infection status by using PCR-based methods.

### Molecular analyses

DNA from the mosquito thorax samples was extracted by using the DNeasy tissue extraction kit combined with the Biosprint96 workstation (QIAGEN), according to the manufacturer’s instructions. A nested-PCR protocol was used to amplify a portion (478-bp long) of the mitochondrial *cytochrome b* gene (mtDNA *cyt b*) of the parasite (see [26,27] for further detailed explanations of the method). PCR-products were purified and sequenced as described by van Rooyen *et al*. [33,34]. We then used MEGA (version 5) for sequence editing and alignment [55]. The MalAvi database allowed us to link genetic polymorphism of the mtDNA *cyt b* gene with previously identified *Plasmodium* lineages [4].

### Statistical analysis

We used multiple linear regression models with the ordinary least squares (OLS) method to investigate whether *C*. *pipiens* density differed between years (2010 and 2011) and between months (April-September). *C*. *pipiens* density (mean egg rafts per container per inspection date, dependent variable) was log (*n*+1) transformed and modeled as a function of year and month of capture nested within year. Mean daily temperature, precipitation and the interaction between temperature and precipitation were considered as continuous covariates in the models. Contrasts between months were then conducted with a Tukey’s HSD test. We used the Pearson’s correlation to investigate covariance pattern between cumulated densities of *C*. *pipiens* and degree-day accumulation.

To assess changes in avian malaria prevalence throughout 2011, we model avian malaria prevalence (proportion of mosquitoes found infected per date) with a quasibinomial error structure as a function of months (April-September). The significance of month was determined using a F-test [56]. Pairwise comparisons between mean monthly prevalence were then conducted with t-tests, using April as the reference month. Sampling dates with less than five collected mosquitoes were discarded from the analysis of prevalence.

We used a Chi-square test to determine whether prevalence of species-specific infection varied during 2011. Adult female mosquitoes caught in September 2011 were dismissed from this analysis as only one mosquito was found infected (over 68 captured). Statistical analyses were conducted using JMP 9.0 (SAS Institute Inc., Cary, NC) and R 2.15.2 [57].

## Results

### *C*. *pipiens* relative abundance

**Figure 1:**
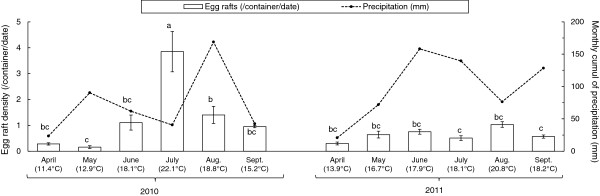
**Seasonal changes in the density of *Culex pipiens *egg rafts and in the rainfall**, **at Dorigny (Switzerland).** Egg raft density was determined as the mean of monthly collected egg rafts per container and per trap date. Error bars are the standard errors of the means. Values between parentheses indicate mean monthly temperature. Egg raft density was significantly different between months not connected by the same letters (Tukey’s HSD test, P < 0.05).

**Figure 2:**
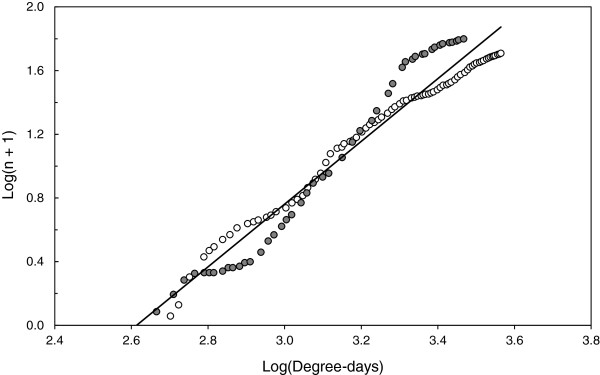
**Relationship between cumulated densities of *Culex pipiens *egg rafts and degree**-**days accumulation.** Densities of *C*. *pipiens* egg rafts (collected egg rafts per container and per collection date) were cumulated throughout collection dates. Cumulated values are presented on a log scale (log(*n* +1)). Degree-days accumulation (log scale) started with the 1^st^ January as biofix date. The regression line (full line) has the following equation y = 1.97× -5.15, R^2^ = 0.95. N= 137 days sampled across seasons (April-September) in 2010 (grey circles) and in 2011 (empty circles).

Egg raft density significantly differed between years (*F*_1,110_ = 26.80; *P* < 0.001) and between months (*F*_10,110_ = 8.53; *P* < 0.001; Figure 1). Egg raft density significantly peaked in July 2010, when environmental conditions were the warmest of the season. No such peak was observed in July 2011, which was exceptionally cold (Figure 1). This pattern resulted in a significant effect of temperature on egg raft density (*F*_1,110_ = 56.58; *P* < 0.001). Indeed, cumulative egg raft density was highly predicted by degree-days accumulation in both years (Pearson’s correlation: 2010: n = 46, *r* = 0.98, *P* < 0.001; 2011: n = 78, *r* = 0.98, *P* < 0.0001; overall: n = 124, *r* = 0.97, *P* < 0.001; Figure 2). Egg raft density was however not significantly influenced by precipitation (*F*_1,110_ = 0.21; *P* = 0.645), neither by the interaction between precipitation and temperature (*F*_1,109_ = 0.98; *P* = 0.325).

### Avian malaria prevalence and lineage diversity

Over 1155 mosquitoes collected across the survey (April-September) 2011, 178 (15.4%) were found positive for avian *Plasmodium* infection (Table 1). Analysis of the mtDNA *cyt b* sequences (430–478 bp) retrieved from mosquitoes’ thorax samples allowed us to identify seven different *Plasmodium* lineages. We found: SYAT05 (50.6% of the infections, n = 90), SGS1 (34.3%, n = 61), AFTRU05 (6.7%, n= 12), GRW11 (4.5%, n =8), PADOM01 (1.7%, n = 3), COLL1 (1.1%, n = 2), PADOM02 (0.6%, n = 1) and one positive sample with undetermined lineage. SYAT05 lineage is associated with the morphospecies *Plasmodium* (*Novyella*) *vaughani* and SGS1, GRW11 and PADOM02 to *Plasmodium* (*Haemamoeba*) *relictum*[58-61]. The remaining lineages AFTRU5, COLL1 and PADOM01, for which morphospecies identities are not yet available in the literature, were grouped as *Plasmodium* spp. lineages.

**Table 1:** **Number of captured female *Culex pipiens *** **and prevalence of avian malaria in the study site**

**Month**	**Number of trap-dates**	**Mean number of traps**	**Mean number of gravid females(/trap/date)**	**N**	**(+)**	**%**
April	9	4	2.6	92	(13)	14.1
May	10	4	9.3	370	(53)	14.3
June	8	3	8.9	214	(28)	13.1
July	8	4	6.2	199	(40)	20.1
August	9	4	5.9	212	(43)	20.3
September	8	2	4.3	68	(1)	1.5
Total				1155	(178)	15.4

Total number of field-caught female *C*. *pipiens* from April to August 2011 (N). The number of PCR-positive mosquito thorax samples (+) and the mean monthly prevalence (%).

### Temporal changes in *Plasmodium* prevalence and lineage community

Avian malaria prevalence significantly varied between months (*F* = 5.79, *P* < 0.001, Table 1). The proportion of infected mosquitoes was relatively stable from April to June (estimate ± SE: May-April: 0.36 ± 0.31, *t* = 1.16, *P* = 0.254; June-April: 0.29 ± 0.33, *t* = 0.86, *P* = 0.393), increased in July (April-July: 0.70 ± 0.32, *t* = 2.161, *P* = 0.037) peaked in August (August-April: 0.71 ± 0.32, *t* = 2.20, *P* = 0.034) before declining drastically in September (September-April: -1.79 ± 0.85, *t* = -2.11, *P* = 0.041) well below the value observed in early spring.

**Figure 3:**
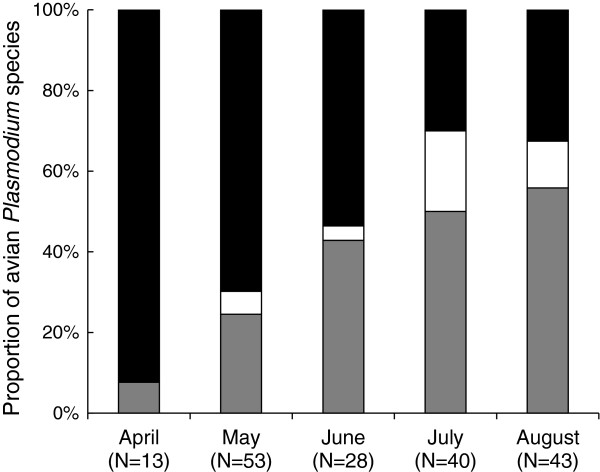
**Changes in avian *Plasmodium *community structure throughout the season (April**-**August) 2011.** Grey bars (*Plasmodium relictum*), black bars (*P*. *vaughani*) and white bars (*Plasmodium* spp.). The total number of infected female *C*. *pipiens* (N) is given for each month.

Prevalence of species-specific infection (*P*. *relictum*, *P*. *vaughani* or *Plasmodium spp*.) significantly differed between months (Chi-square test: n=177, *df* = 8, *χ*^2^ = 35.93, *P* < 0.001). *Plasmodium vaughani* (lineage SYAT05) appeared to be gradually replaced along the season by *P*. *relictum* (lineage SGS1, GRW11 and PADOM02) and later by other *Plasmodium* spp. (COLL1, PADOM1, AFTRU5 lineages) (Figure 3).

## Discussion

### *C*. *pipiens* relative abundance

Year of sampling has a strong effect on *C*. *pipiens* relative abundance. In 2010, the general picture was similar to previous seasonal records conducted in other countries [62-67]. *C*. *pipiens* appear around May and the density slowly increases until a seasonal maximum in July-August [68] or sometimes later in September [54]. In 2011, unusual cold temperatures during summer months may explain the relative low abundance of *C*. *pipiens* over the season survey [65]. The tight relationship between mosquito abundance and field temperatures reported in the present study is well documented in the literature [69-72] and may serve as baseline to model the entomological risk for avian malaria.

### Avian malaria prevalence

The high rate of *C*. *pipiens* infection reported in the present study (16.3%), together with previous surveys conducted at our study site [45], reinforces the view that *C*. *pipiens* is a natural vector of avian malaria in western Switzerland, as observed as well elsewhere in the northern hemisphere [41-45,73]. However, we used highly selective gravid mosquito-traps to target parous *C*. *pipiens* females and our infection rate refers to this group only and was thus not comparable with similar studies using different trapping methods, such as sentinel or light traps.

We found that female *C*. *pipiens* caught in summer (July-August) 2011 were more likely to be infected than those trapped in spring (April-June), a prevalence pattern that is further corroborated by previous field investigations on natural malaria vectors [24,38,39,44]. This result is consistent with the idea that the spring relapse in the bird reservoir hosts results in a seasonal increase of mosquitoes exposed to malaria parasites. Alternatively, evidence that *C*. *pipiens* can adjust their feeding preference for host species as a response to seasonal changes in bird-species abundance is increasing [44,62,74-76]. This process may in turn affect vector prevalence, if the different host species encountered throughout the season are differentially susceptible to avian malaria. Other environmental (abiotic) factors changing seasonally may also have influenced the overall infection rates of *C*. *pipiens*[40].

### *Plasmodium* lineage diversity

*Plasmodium vaughani* (SYAT05 lineage) and *Plasmodium relictum* (SGS1 lineage) were the two most prevalent parasites (50.6% and 34.3%), a result similar to previous surveys conducted across Europe [41,42,45]. Both lineages are probably the most documented parasites in avian-malaria studies, as they have been found nearly worldwide, in hundreds of different bird species [77]. Lineage SGS1 however exploits a wider diversity of bird orders than SYAT05, which is restricted to passerines (Passeriformes). AFTRU5 (*Plasmodium* spp.) was found at a lower prevalence (about 7% of the infections). This lineage has only been found in Blue throats (*Luscinia svecica*) and African thrushes (*Turdus pelios*), in Middle East and West Africa respectively [5,78]. Our study is the first to report its occurrence in Europe. It is possible that lineage AFTRU5 has indeed been imported in Europe by migratory birds. Finally, rare lineages (≤ 4.5% of the infections) included PADOM02 and GRW11 (both attributed to *P*. *relictum*) and COLL1 and PADOM01 of unknown species. These last four lineages are frequently found in native passerines species in Europe [79-83]. It is not yet clear whether these *Plasmodium* lineages were scarce due to rare transmission opportunities at our study site or because they result in high vector mortalities [45].

### Temporal changes in the parasite community structure

A previous study conducted at our study site [45] allowed us to compare the structure of the *Plasmodium* community on a four year interval. *P*. *vaughani* (SYAT05 lineage) and *P*. *relictum* (SGS1, GRW11 and PADOM02 lineages) were found in both studies but other species, such as *P*. *circumflexum* (TURDUS1 lineage) and *P*. *polare* (SW2 lineage) were found only in 2006–2007. On the other hand, lineages AFTRU5 and COLL1 (*Plasmodium spp*.) were new in 2011–2012. To our knowledge, only one study conducted in Japan [37] has previously documented between-year variation in the composition of the avian *Plasmodium* community in vectors: these authors found that the most prevalent *Plasmodium* lineages persist over several years whilst less frequent ones were not consistently encountered at the same period of each year.

In the present study, we also report for the first time that the dominance of *Plasmodium* species within the studied population of mosquitoes varied through the season. Whilst the total prevalence of *Plasmodium* infection, irrespective of strain, increased, infection by *P*. *vaughani* (lineage SYAT05) decreased from spring to summer in favour of *P*. *relictum* (lineage SGS1, GRW11 and PADOM02). This result may be due to seasonal changes in the host feeding preferences of the vectors. Previous studies indeed support the idea that different bird species can host different *Plasmodium* lineages [47,84]. Future studies are needed to investigate temporal changes in (i) the blood-feeding preferences of *C*. *pipiens* and (ii) the communities of *Plasmodium* that infect different bird species in our study system.

An alternative explanation to the seasonal changes in *Plasmodium* lineage composition is that concomitant infection of *C*. *pipiens* by *P*. *relictum* and *P*. *vaughani* may have increased throughout the season, resulting in lower transmissibility of *P*. *vaughani* if vectors had evolved cross-immunity. Blocked transmission of one parasite species by another has for instance been documented in *Aedes aegypti* mosquitoes experimentally co-infected with *P*. *gallinaceum* and *P*. *juxtanucleare*[85]. This process may result in negative periodicity of species-specific infections [86]. Competitive interactions within vectors may also provide an explanation for why we did not find mosquitoes carrying mixed infections.

Finally, different avian *Plasmodium* species may optimally develop within vectors under different environmental conditions. For instance, the minimum temperature requirement for human malaria parasites is 16.5°C, 17.5°C and 18°C for *P*. *malariae*, *P*. *vivax* and *P*. *falciparum* respectively [87] whilst the rodent malaria parasite *P*. *berghei* may be transmitted at lower temperatures [88]. Avian malaria *P*. *relictum* optimally develop within vectors at 27°C [89] and temperatures below 20°C inhibited or strongly delayed sporozoïte development [89,90]. However, the sporogonic cycle of *P*. *vaughani* has been incompletely investigated [77] and further comparative studies at different temperatures are needed.

## Conclusions

We showed that despite an apparent persistence of major avian malaria parasites over several years, the structure of the *Plasmodium* community infecting *C*. *pipiens* does dynamically change, when looking at a finer temporal scale. These changes are most likely due to the interplay of ecological and climatic factors influencing demographic, behavioural and life history parameters of both host and vector populations. Future investigations will determine whether the same changes in the *Plasmodium* lineage composition can repeat over several years and should account for the spatial dimension of parasite, vector and host distributions.

## Competing interests

The authors declare that they have no competing interests.

## Authors’ contributions

FL, OG and PC conceived and designed the study. FL and AD collected the data. FL analysed the data. All authors participated to the writing of the paper. All authors read and approved the final manuscript.

## Authors’ information

OG and PC authors share the senior authorship of the study.

## Supplementary Material

Additional file 1: Figure S1 Relationship between cumulated densities of egg rafts and cumulated densities of gravid C. pipiens females. Densities of egg rafts (mean weekly egg rafts per container per collection date) and densities of gravid female *C*. *pipiens* (mean weekly gravid *C*. *pipiens* per trap per date) were cumulated over the sampling weeks. Cumulated values are presented on a log scale. N = 26 sampling weeks throughout the season survey (April-September 2011). The regression line (grey dotted line) has the following equation y = 0.96× – 1.02 and R2 = 0.99. Click here for file
