# Supplementary material for: Temporal changes in mosquito abundance (Culex pipiens), avian malaria prevalence and lineage composition
Source: Parasit Vectors. 2013 Oct 25;6:307. doi: 10.1186/1756-3305-6-307 (PMC4029311; doi:10.1186/1756-3305-6-307)

Additional files

Additional file 1 - Relationship between cumulated densities of egg rafts and cumulated densities of gravid *C. pipiens* females

Densities of egg rafts (mean weekly egg rafts per container per collection date) and densities of gravid female *C. pipiens* (mean weekly gravid *C. pipiens* per trap per date) were cumulated over the sampling weeks. Cumulated values are presented on a log scale. N = 26 sampling weeks throughout the season survey (April-September 2011). The regression line (grey dotted line) has the following equation y = 0.96x – 1.02 and R^2 = 0.99.

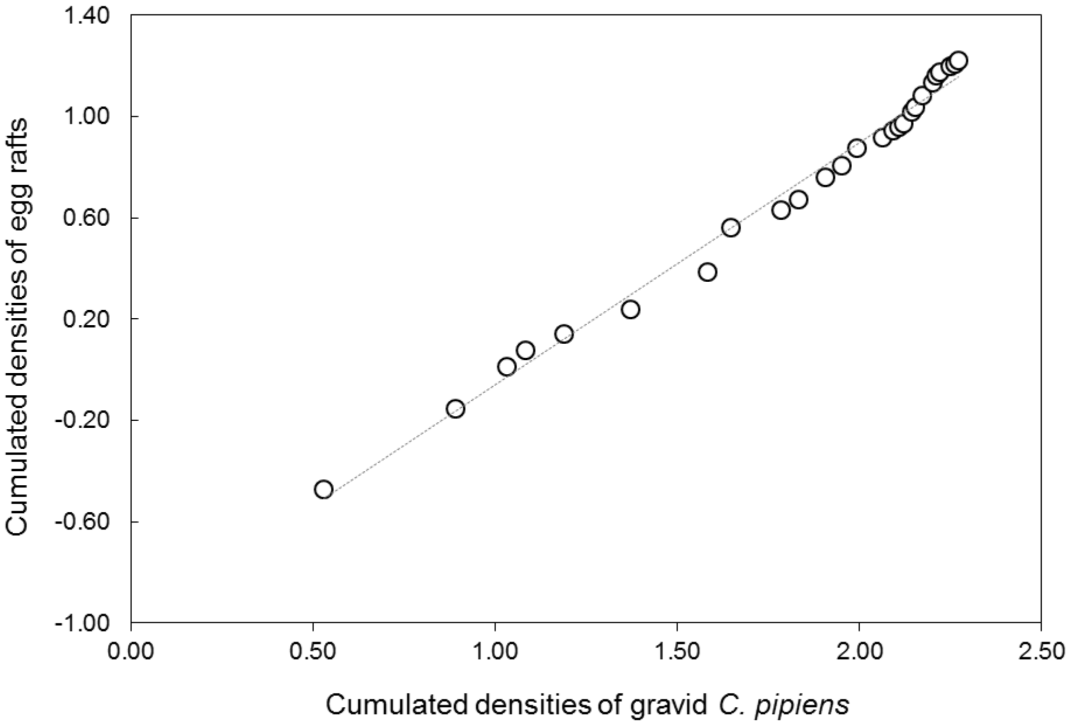

Supplement: Additional file 1: Figure S1 — Relationship between cumulated densities of egg rafts and cumulated densities of gravid C. pipiens females. Densities of egg rafts (mean weekly egg rafts per container per collection date) and densities of gravid female C. pipiens (mean weekly gravid C. pipiens per trap per date) were cumulated over the sampling weeks. Cumulated values are presented on a log scale. N = 26 sampling weeks throughout the season survey (April-September 2011). The regression line (grey dotted line) has the following equation y = 0.96× – 1.02 and R2 = 0.99. [file 1756-3305-6-307-S1.pdf]
